# Association of Physician Referrals with Timely Cancer Care Using Tumor Registry and Claims Data

**DOI:** 10.1089/heq.2021.0089

**Published:** 2022-02-02

**Authors:** Melody K. Schiaffino, James D. Murphy, Vinit Nalawade, Phuong Nguyen, Holly Shakya

**Affiliations:** ^1^ Division of Health Management and Policy, School of Public Health, San Diego State University, San Diego, California, USA.; ^2^ Center for Health Equity, Education, and Research (CHEER), University of California San Diego, La Jolla, California, USA.; ^3^ Department of Radiation Medicine and Applied Sciences, and University of California San Diego, La Jolla, California, USA.; ^4^ Division of Global Health, University of California San Diego, La Jolla, California, USA.

**Keywords:** health care delivery, health disparities, patient–provider communication, communication, cancer and aging, systems science

## Abstract

More Americans are being screened for and more are surviving colorectal cancer due to advanced treatments and better quality of care; however, these benefits are not equitably distributed among diverse or older populations. Differential care delivery outcomes are driven by multiple factors, including access to timely treatment that comes from high-quality care coordination. Providers help ensure such coordinated care, which includes timely referrals to specialists. Variation in referrals between providers can also result in differences in treatment plans and outcomes. Patients who are more often referred between the same diagnosing and treating providers may benefit from more timely care compared to those who are not. Our objective is to examine patterns of referral, or patient-sharing networks (PSNs), and our outcome, treatment delay of 30-days (yes/no). We hypothesize that if a patient is in a PSN they will have lower odds of a 30-day treatment initiation delay. Our observational population-based analysis using the National Cancer Institute (NCI)-linked tumor registry and Medicare claims database includes records for 27,689 patients diagnosed with colorectal cancer from 2001 to 2013, and treated with either chemotherapy, radiotherapy, or surgery. We modeled the adjusted odds of a delay and found 17.04% of patients experienced a 30-day delay in initial treatment. Factors that increased odds of a delay were lack of membership in a PSN (adjusted odds ratio [AOR]: 2.20; 95% confidence interval [CI]: 1.71–2.84), racial/ethnic minority status, and having multiple comorbidities. Provider characteristics significantly associated with greater odds of a delay were if dyads were not in the same facility (AOR: 1.95; 95% CI: 1.81–2.10), if providers were different genders, most notably male (diagnosing) and female (treating) [AOR: 1.23; 95% CI: 1.08–1.40, *p* = 0.0015]. PSNs appear to be associated with reduced of a care delay. The associations observed in our study address the demand for developing multilevel interventions to improve the delivery and coordination of high-quality of care for older cancer patients.

## Introduction

Colorectal cancer (CRC) is the fourth most often diagnosed cancer in American adults (after breast, prostate, and lung cancers).^1^ However, due to screening, diagnostic, and treatment barriers, the inequity in outcomes is glaring; occurring in and killing more Black males than any other subgroup to name on.^2,3^ Five-year survival rates of 70–90% are possible if tumors are detected in early stages, with a drop to ∼17% for later stages.^1^ Survival rates depend on receipt of care that is accessible and timely. Although clinical and patient definitions vary, care coordination refers to the support patients receive as they navigate the medical system to receive timely and high-quality, or guideline concordant, care.^4–8^

Communication between providers within and across health systems is another key element of care coordination that ensures timely care delivery. Communication supports the processing of what can seem like conflicting information for patients as they seek care across the health care continuum.^9,10^ Failure to coordinate care for patients often occurs when patients must move to a different location or level of care intensity (e.g. emergency to inpatient) and there is a lack of communication, or information is not transferred.^11^ Characterizing communication between providers, including referring (diagnosing) physician and a specialist (treating) physician, can provide insights into where we can intervene to improve care transitions and reduce communication barriers for patients.^12^ For this study, we will assess how providers communicate by exploring their referral behavior. Our primary question seeks to explore the role of physician referral behaviors on timely cancer treatment initiation. A secondary question is to explore characteristics associated with physicians that refer, or share, patients.

**FIG. 1.**
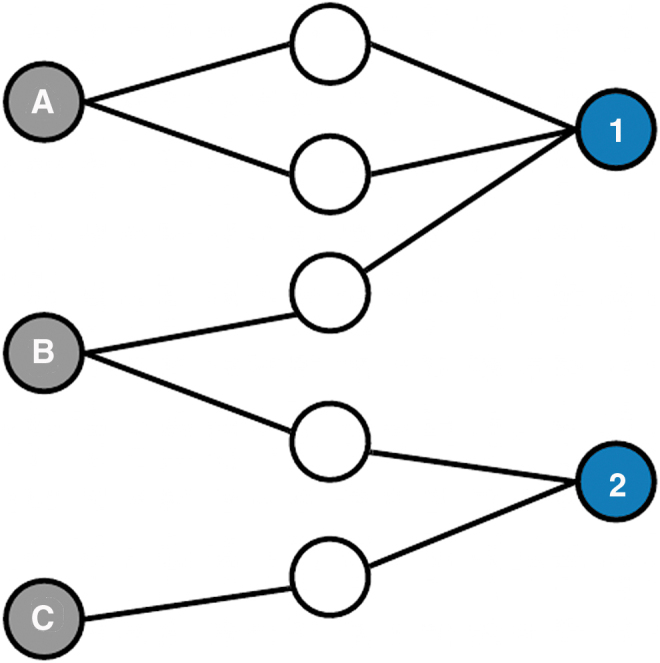
Illustration of PSNs. Diagnosing providers: A, B, C and Treating Providers 1 and 2 share patients indicated by *white circles*. Dyad A-1 shares 2 patients; dyad B1 shares 1; Dyad B2 shares 1; and Dyad C2 shares 1. PSN, patient-sharing network.

The transition from a cancer diagnosis to treatment is critical to ensure the timeliest outcomes. Although the transition from diagnosis to treatment is relatively standardized with an emphasis on timely treatment, especially for later stage patients, barriers to timely care persist. Our primary indicator of this transition is the patient-sharing network (PSN), defined as the number of patients referred between the same diagnosing physician and the same treating physician. To this end we employ network science, an approach used to study relationships between actors (providers). Increasing study in this area is helping to build the evidence of how health care delivery actors (e.g., physicians, providers, and patients) interact with each other and how these interactions are associated with better outcomes.^6,7,13–17^ In the context of providers, these relationships, based on patient referrals, are a measure of the exchange of medical information.^18^ Previous research on physician–physician network ties suggests that when a large number of Medicare patients are shared between two physicians, there will be a higher likelihood that they identify each other as someone with whom they have a professional relationship through either referrals or the exchange of clinical advice thus leading to better outcomes.^18^ In this context, we can consider patients shared between providers, or PSNs, as a potential measure of tie strength, defined as a measure of how close the relationship is between two individuals.^19^ Weak ties can be beneficial for the introduction and diffusion of new ideas, but strong ties are characterized by trust and reciprocity,^20^ characteristics conducive to conscientious communication and problem-solving as are critical to timely care.^21,22^ In this study, we use a measure of tie strength, operationalized by counting the number of shared patients within PSN dyads and divided by the time the dyads have shared those ties. Figure 1 is an example of referrals between three diagnosing providers and two treating provider dyads with different measures of sharing (referral frequency) or tie strength. Only one dyad (A1) would be considered a PSN because they are sharing more than one patient.

We will also explore provider characteristics to assess homophily, a secondary measure of tie strength. Homophily, or sameness between providers, assesses similarity between individuals based upon similar characteristics. Individuals tend to form social ties with people similar to themselves, especially along dimensions of race, gender, and education.^23,24^ We are interested in provider–provider homophily because it increases the likelihood of ties (referrals) between two individuals. Homophily can also contribute to the likelihood of reciprocity and support between actors (providers).^25^ We will characterize provider–provider dyads to explore if delays in care vary by whether or not a patient is in a PSN (dyads with more patients shared/referred) and how the makeup of the PSN is related to these outcomes.^14^

## Methods

The retrospective cohort of CRC patients for this analysis are drawn from the National Cancer Institute (NCI) SEER-Medicare database. These data, obtained through data use agreement and approved for use by both NCI and the UCSD IRB, include 19 national tumor registry databases linked to Medicare beneficiary claims data representing patient health care utilization for the years 2004–2013 (Supplementary Appendix_eFigure 2).^26^ Data were linked to the corresponding administrative claims for Medicare beneficiaries using a unique Patient ID. Provider factors were extracted to characterize dyads from the 2012 American Medical Association (AMA) Physician Masterfile. We utilized the RECORD checklist, an extension of the STROBE guidelines for observational studies; these are included in the Supplementary Appendix.^27^

**FIG. 2.**
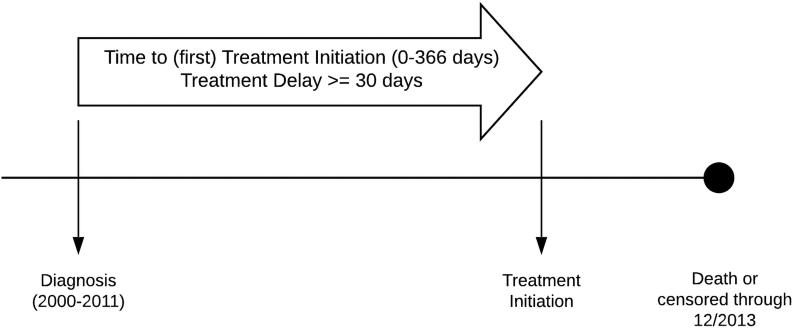
TTI Illustration of time interval under study to develop outcome variable (30-day Delay). TTI, time to treatment initiation.

### Eligibility criteria

Patients were eligible if they were ≥66 years when they were first diagnosed and their time to first treatment (time to treatment initiation [TTI]) occurred within 365 days. The full selection tree figure can be seen in the Supplementary Appendix. We defined CRC using previously published ICD-9 codes.^28^ Additional criteria required the diagnosis of CRC to be a first primary malignant tumor, histologically confirmed, not diagnosed by autopsy or death certificate, and staged as 1 or greater. Cases were first excluded from the base cohort if patients did not have complete data for select demographics (stage, poverty level, *N*=103,026). Finally, we applied a provider-level exclusion whereupon cases were excluded if the information for either the diagnosing physician, pathologist, or treating physician were missing. The final dyad cohort was then restricted to include only patients treated within 365 days (*N*=27,689).

### Outcome variable

We selected a delay of 30 days based on clinical significance and sensitivity analyses of our data using the distribution of treatment initiation within 365 days of diagnosis (Fig. 2). We found gold standard guideline concordant care can range from 14 days to 6 weeks depending on a myriad of factors thus our cutoff ensured adequate coverage. Given the non-normal distribution of the data, we created a dummy outcome variable, with a cut point of treatment initiation >30 days to indicate the presence of a care delay coded as “1,” and no care delay, indicating an ICD code for treatment was identified in ≤30 days, coded as “0” (Reference).

### Primary independent variable

The PSN is a dichotomous variable that indicates patient inclusion in a PSN (Reference) versus not included. We first calculated the number of referrals as an estimate of the average number of Medicare patients shared (referred) diagnosing (dx) and treating (tx) provider dyads for the period between 2004 and 2013 (Medicare FFS share of physician panel) using the following equation^14^:


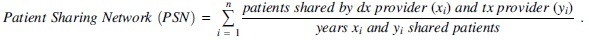


**FIG. 3.**
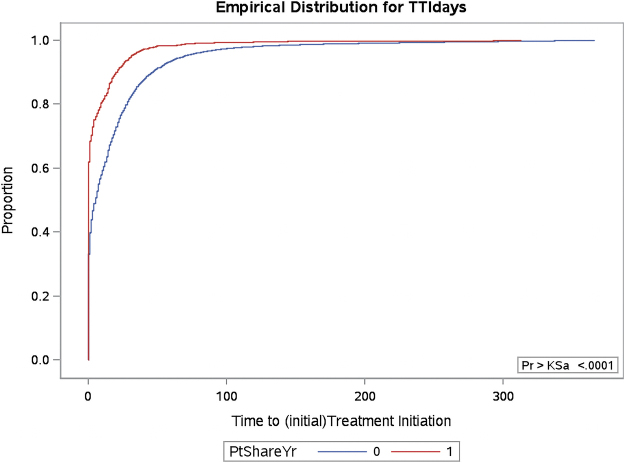
The Kolmogorov-Smirnov (KS) curves compares the number of days it takes each group (PSN=1, non-PSN=0) to initiate treatment. The PSN group (indicated with a red line) demonstrates a larger proportion initiating treatment (in days) sooner than the non-PSN group (blue line). The time each group takes to initial treatment differs significantly as indicated by *p*<.0001. This test confirmed our PSN group cut-offs were appropriate. KS, Kolmogorov-Smirnov (asymptotic test).

We identified *dx* providers using methods adapted from previously published studies that link a diagnosis to pathology confirmation.^7,9,14,18^ The *tx* provider is the provider related to the first treatment (chemotherapy, radiotherapy, or surgery) for CRC after a diagnosis date has been estimated. If the *dx* provider and the *tx* provider are the same person, the dyad was excluded from the analysis since a provider cannot self-share a patient. We used ICD-9 codes for surgery, radiotherapy, and chemotherapy through a previously published approach to extract these data from Medicare claims (Supplementary eAppendix2).^28^ We extracted a count of the average number of patients across each provider dyad. The distribution of this count was also skewed and kurtotic. Using a Kolmogorov–Smirnov asymptotic test to compare different cut points, we selected the top quartile of shared patient categories (Fig. 3). Finally, we created a dichotomous variable to represent those in the top quartile, which included providers who shared (referred) three or more patients, which we coded as “1”=Patients in a PSN (Reference). Those in the bottom three quartiles or sharing fewer than three patients we coded as “0”=Patients not in a PSN. The literature suggests this number can range anywhere from 2 to 10 shared patients.^14,17,28^ Thus, we based our cut points on our data, literature, and expert knowledge of the intensity of cancer care (compared with primary care). We assessed individual patient-level factors in addition to these PSN network (dyad)-level characteristics.

### Patient-level variables

In addition to membership in a PSN, patient factors included age (five categories), gender (1 female; 0 male, Reference), race/ethnicity (white, Black, Hispanic/Latino, Asian, Other), tumor stage (Stages 1, 2, or 3+4), and comorbidity index (Charlson Index CMI; None, 1, and 2 or more). Comorbidity was calculated using the Deyo adaptation for the CMI, with procedure codes that reflect the Romano adaptation without cancer as an established approach in cancer research.^29–31^

### Provider network-level variables

Provider dyads were categorized as having homophily, or being similar, if they matched on gender, colocation, and discipline or specialty. We recorded dyad gender concordance and discordance with the male–male provider dyad serving as the reference group given the dominance of male gender in oncology.^32,33^ Primary specialties were categorized as oncology, nononcology, or primary care (Reference). Colocation was coded as “1” if either provider in a dyad did not share a facility code and “0” if they did (Reference) using facility codes from physicians from the AMA file.

### Analysis

We calculated an inverse propensity weight after sensitivity analysis detected differences in cohorts for patients that did and did not have missing observations for the inclusion criteria: age at diagnosis, gender, race/ethnicity, marital status, poverty indicator, CMI (comorbidity), physician factors, and cancer stage. We stratified the frequencies across the PSN values but only for the descriptive analysis, inferential models were not stratified due to sample size and SEER-Medicare minimum reporting restrictions. A multivariable logit regression was modeled to compare the effect of provider and patient-level factors associated with odds of a care delay. All analyses were conducted using SAS 9.4 (Cary, NC).

## Results

Average TTI was 17.3 days (SD 34.6) for all Medicare patients diagnosed with CRC within the 1-year study period. The average number of shared patients (referrals) in a PSN was 1.2 patients (SD 0.47, median 1.0 patients) per year under study. Only 4.8% of patients were in a PSN (three or more shared patients) representing the top quartile of referrals between providers.

### Descriptive results

Care delays differed significantly by PSN inclusion; we found 17.60% of patients not in a PSN experienced a care delay, whereas only 5.30% of patients in a PSN (*p*<0.0001) were delayed. This pattern persisted across all covariates in the PSN group at both the patient and provider levels, though not all were statistically significant. Table 1 details some of these results, additional table details are masked due to small cell size in compliance with NCI data-sharing guidelines.

**Table 1.** Patient- and Network-Level Characteristics of Older Patients with Colorectal Cancer that do Not Experience a 30-Day Treatment Delay by PSN Status in SEER-Medicare (*N*=27,689)

	PSN (3 patients shared)		No PSN (≤2 patients shared)	
Patient-level factors (all values are column %)	No care delay	*p* ^a^	No care delay	
	*n* (94.7%)		*n* (82.4%)	*p* ^a^
SEER cancer stage				
Stage 1	528 (43.5)	0.1777	7982 (36.7)	**<.0001**
Stage 2	521 (42.9)		9257 (42.6)	
Stage 3 | 4	165 (13.6)		4518 (20.8)	
Comorbidities				
No comorbidities	754 (62.1)	0.3999	12794 (58.8)	**0.0236**
1 comorbidity	301 (24.8)		5600 (25.7)	
2 or more (MCC)	159 (13.1)		3363 (15.5)	
Patient gender				
Male	524 (43.2)	0.8771	9454 (43.5)	**<.0001**
Female	690 (56.8)		12303 (56.6)	
Patient age				
66–70 years	193 (15.9)	0.9752	3494 (16.1)	**<.0001**
71–75 years	256 (21.1)		4687 (21.5)	
76–80 years	345 (28.4)		5165 (23.7)	
81–85 years	250 (20.6)		4646 (21.4)	
85+ years	170 (14.0)		3765 (17.3)	
Patient race/ethnicity				
White	>1107 (>91.0)	**0.0153**	18832 (86.6)	**0.0046**
Asian	<11 (<1.0)		682 (3.1)	
Black	80 (6.6)		1491 (6.9)	
Hispanic/Latino	<11 (<1)		271 (1.3)	
Other	16 (1.3)		482 (2.2)	
Patient marital status				
Single	71 (5.9)	0.0518	1736 (8.0)	**0.0001**
Divorced	44 (3.6)		1203 (5.5)	
Married	636 (52.4)		10578 (48.6)	
Other	463 (38.1)		8240 (37.9)	
Patient poverty status				
0% to <5%	382 (31.5)	0.9968	6769 (31.1)	**0.0092**
5% to <10%	330 (27.2)		6138 (28.2)	
10% to <20%	330 (27.2)		5459 (25.1)	
20% to 100%	172 (14.2)		3391 (15.6)	
**Provider-Network Factors**				
DX-TX physician dyad gender				
Male–male	1152 (>94.0)	**0.0008**	19097 (87.8)	**<.0001**
Female–female	58 (4.8)		464 (2.1)	
Male–female	<11 (<1.0)		1121 (5.2)	
Female–male	<11(<1.0)		1075 (4.9)	
Dyad co-located (facility)				
DX and TX not c-located	512 (42.2)	0.1315	15269 (70.1)	**<.0001**
DX and TX colocated	702 (57.8)		6488 (29.8)	
Diagnosing (DX) provider primary specialty				
Primary care	44 (3.6)	0.5101	3901 (17.9)	**<.0001**
Any nononcology	>1159 (>96.0)		17577 (80.9)	
Oncology (med/rad/surg)	<11 (<1.0)		263 (1.2)	
Treating (TX) provider primary specialty				
Primary care	<11 (<1.0)	**0.0356**	341 (1.6)	**<.0001**
Any nononcology	>1187 (>98.0)		19607 (90.1)	
Oncology (med/rad/surg)	16 (1.3)		1805 (8.3)	

Bold indicates statistical significance at *p*<0.05 or *p*<0.0001.

^*^p-value <.0001; ^**^The values masked by ‘^**^’ and the < or > estimates are presented in compliance with the CMS cell size suppression policy minimum threshold for the display of CMS data (https://resdac.org/articles/cms-cell-size-suppression-policy).

^a^ Fishers exact p-value calculated and reported for crosstabs where cell values were less than *n*=5.

At the patient level, patients with cancer stage 3 or 4 reported the lowest proportion of care delay; however, this was only significant in the non-PSN group (14.58%, *p*<0.0001 vs. PSN 2.37%, *p*=0.1777). Patients with one comorbidity were most likely to experience care delays compared with patients with none or multiple (2 or more) conditions, again this was only significant in the non-PSN group (18.69%, *p*=0.0236 vs. 18.14%, *p*=0.3999). The youngest patients (66–70) were more likely to experience a care delay (vs. no delay; *p*<0.0001). Conversely, Asian patients were the group with the highest frequency of a care delay in the PSN group (36.36%, *p*=0.0153), Hispanic/Latino patients were the highest in the non-PSN group (22.79%, *p*=0.0046).

At the PSN provider-network level, we found a higher frequency of delays among mixed-gender physician dyads versus same-gender dyads, interestingly this was inverted for discordant PSN (female–male, *p*=0.0008) and non-PSN (male–female, *p*<0.0001) groups. Dyad colocation resulted in reduced care delays in both groups (PSN 4.38%, *p*=0.0019 vs. non-PSN 11.05%, *p*<0.0001). Only treating physician specialty was significant across both PSN and non-PSN groups.

### Multivariable results

Overall, we found that patients not in a PSN experienced over twofold greater odds of a 30-day delay compared with patients in a PSN (adjusted odds ratio [AOR]: 2.20; 95% confidence interval [CI]: 1.71–2.84; <0.0001). Table 2 details patient and PSN network factors and their association with a care delay.

**Table 2.** Multivariable Odds of Older Patients with Colorectal Cancer that Experience a 30-Day Treatment Delay in SEER-Medicare (*C*=0.75; *N*=27,689)

Patient-level	30 day treatment (care) delay		
	AOR	95% CI	*p*
SEER tumor stage (at diagnosis)			
Stage 1	Ref.	—	
Stage 2	0.74	0.55–0.99	**0.0411**
Stage 3 and 4	0.5	0.44–0.56	**<.0001**
Comorbidities			
No comorbidities	Ref.	—	
1 comorbidity	1.18	1.08–1.28	0.0001
2 or more (MCC)	1.16	1.03–1.30	**0.0158**
Patient gender (SEER)			
Male	Ref.	—	**0.0068**
Female	0.89	0.82–0.97	
Patient age			
66–70 years	Ref.	—	
71–75 years	1.05	0.94–1.17	0.4238
76–80 years	1.08	0.95–1.23	0.2153
81–85 years	1.03	0.88–1.19	0.7333
85+ years	0.98	0.82–1.18	0.8614
Patient race/ethnicity			
White	Ref.	—	
Asian	1.09	0.90–1.33	0.3881
Black	1.46	1.25–1.72	<.0001
Hispanic/Latino	1.43	1.09–1.90	**0.0113**
Other	1.09	0.87–1.38	0.4428
Patient marital status			
Single	Ref.	—	
Divorced	1.11	0.91–1.35	0.3158
Married	0.94	0.82–1.09	0.4257
Other	0.95	0.83–1.09	0.4466
Patient poverty level			
0% to <5%	Ref.	—	
5% to <10%	1.09	0.90–1.33	0.3631
10% to <20%	1.1	0.80–1.52	0.569
20% to 100%	1.06	0.73–1.54	0.7661
**Provider network-level**	**AOR**	**95% CI**	***p***
Patient sharing network (PSN)			
Yes (3 patients shared)	Ref.	—	
No (2 patients shared)	2.2	1.71–2.84	<.0001
Gender of diagnosing and treating provider dyads			
Male–male	Ref.	—	
Female–female	0.66	0.48–0.89	**0.0063**
Female–male	1.02	0.87–1.19	0.8285
Male–female	1.23	1.08–1.40	**0.0015**
Diagnosing and treating physician dyads Co-located (Facility)			
DX and TX colocated	Ref.	—	
DX and TX not colocated	1.95	1.81–2.10	<.0001
Diagnosing physician (DX) primary specialty			
Primary care	0.61	0.59–0.72	**<.0001**
Any nononcology specialty	Ref.	—	
Oncology (med/rad/surg)	0.4	0.29–0.55	**<.0001**
Treating physician (TX) primary specialty			
Primary care	0.61	0.50–0.73	**<.0001**
Any nononcology specialty	0.14	0.12–0.15	**<.0001**
Oncology (med/rad/surg)	Ref.	—	

Bold indicates statistical significance at *p*<0.05 or *p*<0.0001.

AOR, adjusted odds ratio; CI, confidence interval.

Patient factors that resulted in significantly lower odds of a care delay included those at a later stage (stage 2, AOR: 0.74 and stage 3 or 4, AOR: 0.50) compared with those at stage 1 (Reference). Female patients experienced lower odds of a care delay compared with males (AOR: 0.89; 95% CI: 0.82–0.97; *p*=0.0068). Conversely, one or multiple comorbidities resulted in 18% and 16% significantly greater odds of a delay, respectively, compared with patients with no comorbidities (Reference). We observed significantly greater odds of a delay for Black and Hispanic/Latino patients, 46% and 43%, respectively, compared with white patients (AOR Latino 1.43; *p*=0.0113; AOR Black 1.46; *p*<0.0001).

In addition to PSNs, we observed additional provider-level factors. PSN dyads with female–female providers reported significantly lower odds of a care delay (AOR: 0.66; 95% CI: 0.48–0.89, *p*=0.0063) compared with male–male dyads (Reference). The inverse was true for male–female dyads, with 23% greater odds of a care delay (*p*=0.0015). Dyad colocation was significant when providers were not in the same facility, the odds of a delay were 95% higher compared with the reference group, both providers were colocated in a facility (AOR: 1.95; 95% CI: 1.81–2.10, *p*<0.0001).

## Discussion

Our study findings support our hypothesis that physician PSNs (referrals) may be an opportunity to address care delays, particularly for referring older or sicker patients. In addition to observing higher odds of a delay among patients seen by physicians who did not share patients, we also observed significantly higher odds of a delay among Black and Hispanic/Latino patients and those with more comorbid conditions.

Our finding that physicians who were not colocated contributed to significantly higher odds of a care delay aligns with the growing evidence on the role of location in care access.^34^ Place is often a barrier to care for rural and minority communities as distance to a provider can also influence care coordination and timely care.^35,36^ We also found that the gender configuration of the physician dyads played a role in the time it takes to begin treatment, another important insight into how the interpersonal dynamics of physician–physician relationships impact outcomes. Provider patient-sharing relationships have the potential to help understand transitions between physicians that can contribute to our understanding of a patient's clinical journey. Our findings that increased patient-sharing is associated with improved outcomes align with growing evidence that suggest exploring the referral and other behaviors (interactions) between providers holds potential for improving care quality and overall outcomes for patients.^15,37^ We also find in this study that patient-sharing itself, although predictive, does not tell the whole story. Our colocation findings suggest that doctors in the same facility may be part of a more socially integrated medical community, with increased opportunities for interaction in person and interpersonal accountability.

Patients treated by female–female dyads had the lowest odds of a 30-day delay, and those treated by male diagnosing physicians and female treating physicians had the highest compared with male–male dyads. This finding is salient in a field that remains male dominated.^38^ Previous research suggests patients treated by female internists and surgeons have better outcomes than those treated by male doctors because female doctors may be more likely to take a patient-centered approach and follow evidence-based guidelines.^39^ The research suggests that two female doctors who work collaboratively will communicate more effectively than when one of the providers is male. Another element of our study is the difference in the male–female dyads in comparison with others. Female physicians were a clear minority in the sample. It is possible that in a status-oriented field such as medicine, male-diagnosing physicians are not able to communicate as effectively with female physicians who are higher in status to them, or that female treating physicians may not be as receptive to communication from male physicians. One finding suggests that the decision to refer a patient is largely driven by gender, and that there may a preference for a male physician when males make the referral due to homophily, the same driver of our patient-sharing outcome.^24^ This dynamic warrants further research on dyad gender dynamics and how they can be managed effectively to improve patient outcomes.

Given the underlying assumptions of network science and cross-sectional research, our study has limitations. Our methods draw from existing studies that helped us to identify diagnosing providers; however, it remains challenging given the fragmentation in administrative data. Medicare coverage only begins for our sample at age 65, so we have little knowledge of patient health services and quality of care coordination before this age, this is also an issue with care occurring before a patient's cancer diagnosis. Our variable of colocation is limited to location of providers in a facility; although this does provide a good estimate of care coordination within a system, it excludes nearby providers that may be more realistic of referral behaviors. This is especially the case for rural and low-mobility Medicare beneficiaries. We further limited our data set to create dyads, thus results are not generalizable outside of our weighted Medicare population. It is important to mention that the sample was still sizable, and our estimates were significant despite our conservative data management which is encouraging given the exploratory nature of this study.

## Conclusion

Issues of equity can manifest not only *between* providers when making referral decisions but can also occur *among* individual providers resulting in differential treatment decisions. Understanding individual provider behaviors in addition to their provider-to-provider behaviors and patterns can lead to additional contributions to the field.

Another potential focus of study to consider in this area is the role of psychosocial benefits to providers. This group faces multiple constraints on their attention, time, and focus. For patients, the next step is to determine if this approach works for other cancer sites and whether there are survival benefits in addition to those observed in this study. In 2001, the Institute of Medicine (IOM) published a report underscoring the importance of high-quality care delivery.^40^ For the past two decades, this report has been the framework for evolving recommendations that emphasize the growing demand for harnessing our massive health care data resource.^40–42^ Our analysis leverages a massive database that links national tumor registry data with administrative claims to gain insights into how provider-to-provider referral behaviors and location affect care delivery, an area that to date has been poorly studied and can have serious implications for equitable care delivery. Focusing on the multilevel interactions at the provider and patient levels of care delivery can support more equitable systems of care to promote the delivery of more equitable and timely care for all patients.

## Supplementary Material

Supplemental data

## Abbreviations Used

AMA: American Medical Association
AOR: adjusted odds ratio
CI: confidence interval
CRC: colorectal cancer
IOM: Institute of Medicine
KS: Kolmogorov-Smirnov (asymptotic test)
NCI: National Cancer Institute
PRF: physician referral frequency
PSN: patient-sharing network
SD: standard deviation
TTI: time to treatment initiation
