# Supplementary material for: Association of Physician Referrals with Timely Cancer Care Using Tumor Registry and Claims Data
Source: Health Equity. 2022 Feb 2;6(1):106–15. doi: 10.1089/heq.2021.0089 (PMC8896170; doi:10.1089/heq.2021.0089)
Supplement: Supplemental data [file Suppl_Appendix.docx]

Supplemental online content for:

Can physician referrals reduce care delays for colorectal cancer patients?

**APPENDIX LIST**

**eAppendix1.** Supplemental Methods

**eAppendix2.** Medicare claim Codes

**eTable1.** Patient and Network Level Characteristics of SEER-Medicare Older Adult CRC Patients that Experience a 30-Day Treatment Delay (N=27689).

**eFigure 1.** Patient Diagnosis and Treatment Decision

**eFigure 2.** Patient-level and Provider-Level Network Case Selection Tree. Flow chart of algorithm development using RECORD (an adapted expansion on STROBE guidelines).

**eFigure 3.** Histogram of Diagnosing (DX) Provider-Treating (TX) Provider Dyad by PRF dyad and Time to Treatment (in days). A PRF is associated with shorter time to treatment.

**eAppendix1. Supplemental Methods**:

This study is a retrospective secondary cohort of colorectal cancer (CRC) cases using SEER-Medicare linked data. SEER-Medicare consists of 19 national tumor-registry databases linked to Medicare beneficiary claims data representing the years 2000-2013^1^ SEER includes U.S. Census estimates from 1990-2000 in addition to detailed tumor diagnosis and treatment information from 19 cancer registries across the U.S. Medicare claims contain patient visit data for eligible older adults regarding diagnoses and procedures performed as well as referral characteristics.^1^ SEER-Medicare data were linked to the corresponding administrative claims for Medicare beneficiaries using their Patient ID number. Finally, we extracted provider demographics from the 2012 American Medical Association’s (AMA) Physician Masterfile. The AMA Masterfile is a database with sociodemographic, medical training, and current practice data. The providers in this database are identified by their National Provider Identifier (NPI) or Unique Provider Identification Number (UPIN). The Masterfile was linked via the encrypted NPI or UPIN, obtained from the claims data, to extract diagnosing and treating dyads. This allowed us to examine patient-sharing relationships between providers and explore characteristics about both patients and providers.

Eligibility Criteria. Patients were included if their age was not missing and they were 66 or older when they had a first diagnosis of CRC, the full selection tree can be seen in Figure 2. We defined CRC using ICD9 codes (See Supplementary File).^2^ The diagnosis of CRC had to be a first primary malignant tumor, histologically confirmed, not diagnosed by autopsy or death certificate only, and staged as 1 or greater (unstaged cases were excluded). Cases were excluded if the month of diagnosis was missing from the SEER registry data. We selected cases with continuous Medicare coverage for Parts A and B without Part C covered from one year before diagnosis until the last follow-up or date of death. This was done to ensure that comorbidities could be captured from one year before diagnosis up to one month before diagnosis.

Comorbidity was calculated using the Deyo adaptation of the Charlson comorbidity index, with several procedure codes that reflect the Romano adaptation without cancer as an established approach.^3-6^ Cases were also excluded from the base cohort if patients did not have complete data for select demographics (SEER stage, SEER reported poverty level, N = 103,026). Finally, cases were excluded if the information for either the diagnosing physician, pathologist, or treating physician was missing. The final dyad cohort was then restricted to patients with time to treatment initiation of 366 days or less (N = 27,689).

*Outcome/Dependent Variable.* We transformed our outcome variable from a continuous count variable to a dichotomous measure for descriptive and multivariable regression models. For diagnostic analyses, Time to treatment (TTI) is a continuous estimate of the time interval, in days, between the date of cancer diagnosis and the date of the first cancer-related treatment. For the The value was extracted from the administrative claims linked to SEER cases. The first treatment for CRC after diagnosis was calculated using codes for surgery, radiotherapy, and chemotherapy using an approach previously used to extract TTI from claims (See Supplementary Files).^2^ Initially extracted as an ordinal count variable, we assessed its distribution (mean, median, quartiles) and found the distribution to be skewed and kurtosis as is common for this type of data (See Fig.3 ). We created a binary outcome variable, with a cut point of greater than 30 days was a delay in TTI or equal to “1”, and less than or equal to 30 days was equal to “0”, or no delay in TTI, our reference group.

*Primary Independent Variable*: Provider tie strength or Provider Referral Frequency (PRF). Our primary independent variable of interest is the average annual shared patients from the time period between 2000-2013 (Medicare FFS share of the physician panel) between a diagnosing and treating physician .^7^ After extracting the outcome variable, we applied limitation criteria for provider-level relationships. The availability of provider characteristics to calculate patient-sharing, was based the presence of provider identifiers in *both* the SEER-Medicare and 2012 National Provider Database files (See Fig. 2)

PRF, our primary independent variable, is the average count of shared patients per year under study between the diagnosing and treating provider dyads (2000-2013). Provider dyads contain the count of patients referred by the same diagnosing and treating providers divided by the number of years they share patients. If the dyad was made up of the same diagnosing and treating provider, the observation was dropped from the analysis as a provider cannot self-share a patient.

We identified diagnosing providers using methods adapted from previously published studies.^7-10^ For our study, we cross-referenced providers referring patients to pathologists for diagnostic confirmation, limited to the closest date (before) the first treatment date. Treating providers were those providers performing a first cancer-specific treatment (chemotherapy, radiation, surgery, or some combination) based on an identified list of codes for treatment.^2^

PRF for our descriptive and inferential analyses was coded as a dichotomous variable. After assessing the count variable we selected the top-quartile as our cut-point due to the distribution of the data. For analysis we defined a PRF as not present and coded it as “1” if tie-strength was <=2 patients and PRF was present and coded as “0” if 3+ shared-patients were observed (reference group).

For our study, we cross-referenced providers referring patients to pathologists for diagnostic confirmation, limited to the closest date (before) the first treatment date. Treating providers were those providers performing a first cancer-specific treatment (chemotherapy, radiation, surgery, or some combination) based on an identified list of codes for treatment.^2^

**eAppendix2.** Medicare claim Codes

**Diagnostic Codes:**

NCI Codes for CRC

ICD9 /WHO 2008 site recodes (21041, 21042, 21043, 21044, 21045, 21046, 21047, 21048, 21049, 21051, and 21052)

**Surgical Codes:**

HCPCS '44140', '44141', '44143', '44144', '44145', '44146', '44147', '44150', '44151', '44152', '44153', '44155', '44156', '44160', '44110', '44111', '44112', '44113', '44114', '44116', '44119', '44123', '44160', '44170', /*Colostomy/Ileostomy per 30 days*/'44310', '44316', '44320', '44322', '44340', '44345', '44620', '44625', '44626'

AND

Icd9 '688', '4571', '4572', '4573', '4574', '4575', '4576', '4579', '458', '484', '4841', '4849', '485', '4861', '4862', '4863', '4864', '4865', '4869', '4601', '4603', '4610', '4611', '4613', '4614', '4620', '4621', '4622', '4623', '4624', '4631', '4639', '4640', '4641', '4643', '4650', '4651', '4652'

**Radiation**:

HCPCS: '76370', '76950', '77261', '77262', '77263', '77280', '77285', '77290', '77295', '77299',

'77300', '77301', '77305', '77310', '77315', '77321', '77326', '77327', '77328', '77331', '77332', '77333', '77334', '77336', '77370', '77399', '77401', '77402', '77403', '77404', '77406', '77407', '77408', '77409', '77411', '77412', '77413', '77414', '77416', '77417', '77427', '77431', '77432', '77470', '77499', '77520', '77523', '77750', '77761', '77762', '77763', '77776', '77777', '77778', '77781', '77782', '77783', '77784', '77789', '77790', '77799',

IDC-9: '9221', '9222', '9223', '9224', '9225', '9226', '9227', '9228', '9229', '9230', '9231', '9232', '9233', '9239')

**Chemotherapy:**

HCPCS: '36260', '96400', '96405', '96406', '96408', '96410', '96412',

'96414', '96420', '96422', '96423', '96425', '96440', '96445', '96450', '96520', '96530', '96542', '96545', '96549', '95990', '95991', 'A4301', 'E0782', 'E0783', 'E0784', 'E0785', 'E0786', 'G0355', 'G0357', 'G0358', 'G0359', 'G0360', 'C9411', 'J0207', 'J0640', 'J0880', 'J1190', 'J1440', 'J1441', 'J1950', 'J9217', 'J9218', 'J9219', 'J2405', 'J2430', 'J2505', 'J2820', 'J3487', 'J8520', 'J8521', 'J8530', 'J8560', 'J8565', 'J8600', 'J8610', 'J8700', 'J8999', 'K0415', 'KO416', 'Q0083', 'Q0084', 'Q0085', 'Q0136', 'Q0137', 'Q0179', 'S0177', 'S0181'

Physician Specialties: (Primary or Secondary) Obtained from 2012 AMA Masterfile

"ATP", "CLP", "DMP", "FOP", "HMP", "MGG", "MGP", "NP", "PCH", "PCP", "PP", "PTH", "SP"

REFERENCES

1. SEER-MEDICARE. In. *Surveillance, Epidemiology, and End Results (SEER) Program Populations (1969-2017)* December 2016 ed. National Cancer Institute: DCCPS, Surveillance Research Program; 2019.

2. Pruitt SL, Harzke AJ, Davidson NO, Schootman M. Do diagnostic and treatment delays for colorectal cancer increase risk of death? *Cancer Causes Control.* 2013;24(5):961-977.

3. Charlson ME, Pompei P, Ales KL, MacKenzie CR. A new method of classifying prognostic comorbidity in longitudinal studies: development and validation. *J Chronic Dis.* 1987;40(5):373-383.

4. Deyo RA, Cherkin DC, Ciol MA. Adapting a clinical comorbidity index for use with ICD-9-CM administrative databases. *J Clin Epidemiol.* 1992;45(6):613-619.

5. Romano PS, Roos LL, Jollis JG. Adapting a clinical comorbidity index for use with ICD-9-CM administrative data: differing perspectives. *J Clin Epidemiol.* 1993;46(10):1075-1079; discussion 1081-1090.

6. Klabunde CN, Potosky AL, Legler JM, Warren JL. Development of a comorbidity index using physician claims data. *J Clin Epidemiol.* 2000;53(12):1258-1267.

7. Landon BE, Keating NL, Barnett ML, et al. Variation in patient-sharing networks of physicians across the United States. *JAMA.* 2012;308(3):265-273.

8. Pollack CE, Lemke KW, Roberts E, Weiner JP. Patient sharing and quality of care: measuring outcomes of care coordination using claims data. *Med Care.* 2015;53(4):317-323.

9. Pollack CE, Wang H, Bekelman JE, et al. Physician Social Networks and Variation in Rates of Complications After Radical Prostatectomy. *Value in Health.* 2014;17(5):611-618.

10. Barnett ML, Landon BE, O'Malley AJ, Keating NL, Christakis NA. Mapping physician networks with self-reported and administrative data. *Health Serv Res.* 2011;46(5):1592-1609.

**eTable1.** Patient and Network Level Characteristics of SEER-Medicare Older Adult CRC Patients that Experience a 30-Day Treatment Delay (N=27689).

| **Patient-Level Factors** | |  |  | |
| --- | --- | --- | --- | --- |
|  |  | **Total**  N | **Care Delay**  n (%) | **NO Delay**  n (%) |
|  |  |  | 4718 (17.04) | 22971 (82.96) |
| SEER cancer stage | Stage 1  Stage 2  Stage 3 \| 4 | 10341  11890  5458 | 1831 (17.71)  2112 (17.76)  775 (14.20) | 8510 (82.29)  521 (82.24)  165 (85.80) |
| Multiple Comorbidities | No comorbidities  1 comorbidity  2 or more (MCC) | 16244  7209  4263 | 2696 (16.60)  1308 (18.14)  714 (16.86) | 13548 (83.40)  5901 (81.86)  3522 (83.14) |
| Patient Gender (SEER) | Male  Female | 12241  15448 | 2263 (18.49)  2455 (15.89) | 9978 (81.51)  12993 (84.11) |
| Patient Age | 66-70yrs  71-75yrs  76-80yrs  81-85yrs  85+yrs | 4553  6082  6706  5797  4551 | 866 (19.02)  1139 (18.73)  1196 (17.83)  901 (15.54)  616 (13.54) | 3687 (80.98)  4943 (81.27)  5510 (82.17)  4896 (84.46)  3935 (86.46) |
| Patient Race/Ethnicity | White  Asian  Black  Hispanic/Latino  Other | 23931  851  1940  357  610 | 3995 (16.69)  162 (19.04)  369 (19.02)  80 (22.41)  112 (18.36) | 19936 (83.31)  689 (80.96)  1571 (80.98)  277 (77.59)  498 (81.64) |
| Patient Marital Status | Single  Divorced  Married  Other | 2175  1526  13655  10333 | 368 (16.92)  279 (18.28)  2441 (17.88)  1630 (15.77) | 1807 (83.08)  1247 (81.72)  11214 (82.12)  8703 (84.23) |
| Patient Poverty Status | 0%-<5%  5% to <10%  10% to <20%  20% to 100% | 8714  7816  6924  4235 | 1563 (17.94)  1348 (17.25)  1135 (16.39)  672 (15.87) | 7151 (82.06)  6468 (82.75)  5789 (83.61)  3563 (84.13) |
| **Physician Network Factors** | |  |  |  |
| Patient Referral Frequency | Yes (≥3 Patients Shared)  No (≤2) | 1282  26407 | 68 (5.30)  4650 (17.61) | 1214 (94.70)  21757 (82.39) |
| Diagnosing-Treating Physician Dyad Sex | Male-Male  Female-Female  Male-Female  Female-Male | 24183  576  1600  1330 | 3934 (16.27)  54 (9.38)  479 (29.94)  251 (18.87) | 20249 (83.73)  522 (90.63)  1121 (70.06)  1079 (81.13) |
| Provider Dyad Co-Location | DX and TX **not** collocated  DX and TX collocated | 19653  8036 | 3872 (19.70)  846 (10.53) | 15781 (80.30)  7190 (89.47) |
| Diagnosing (DX) Provider Primary Specialty | Primary Care  Any Non-Onc  Any Oncology (Med/Rad/SurgOnc) | 4585  22769  314 | 640 (13.96)  4022 (17.66)  51 (16.24) | 3945 (86.04)  18747 (82.34)  263 (83.76) |
| Treating (TX) Provider Primary Specialty | Primary Care  Any Non-Onc  Any Oncology (Med/Rad/SurgOnc) | 552  23470  3661 | 208 (37.68)  2668 (11.37)  1840 (50.26) | 344 (62.32)  20802 (88.63)  1821 (49.74) |

Abbreviations: SEER, Surveillance, Epidemiology, and End Results; CRC, colorectal cancer; Med/Rad/SurgOnc, Chemotherapy/Radiotherapy/SurgicalOncology


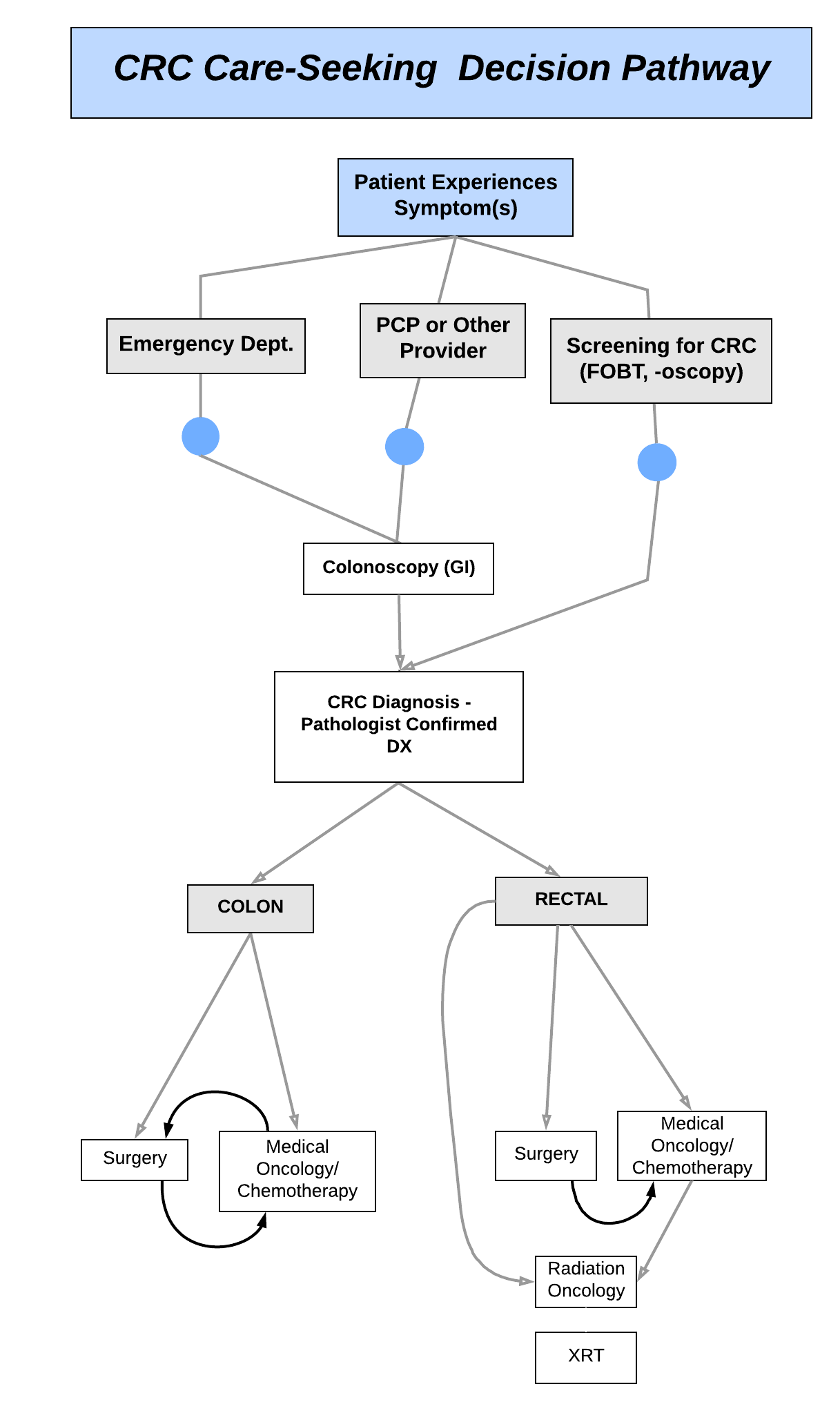


**eFigure 1.** Patient Diagnosis and Treatment Decision

Abbreviations: CRC, colorectal cancer; PCP, Primary Care Physician ; FOBT, Fecal Occult Blood Test; DX, Diagnosis.

**
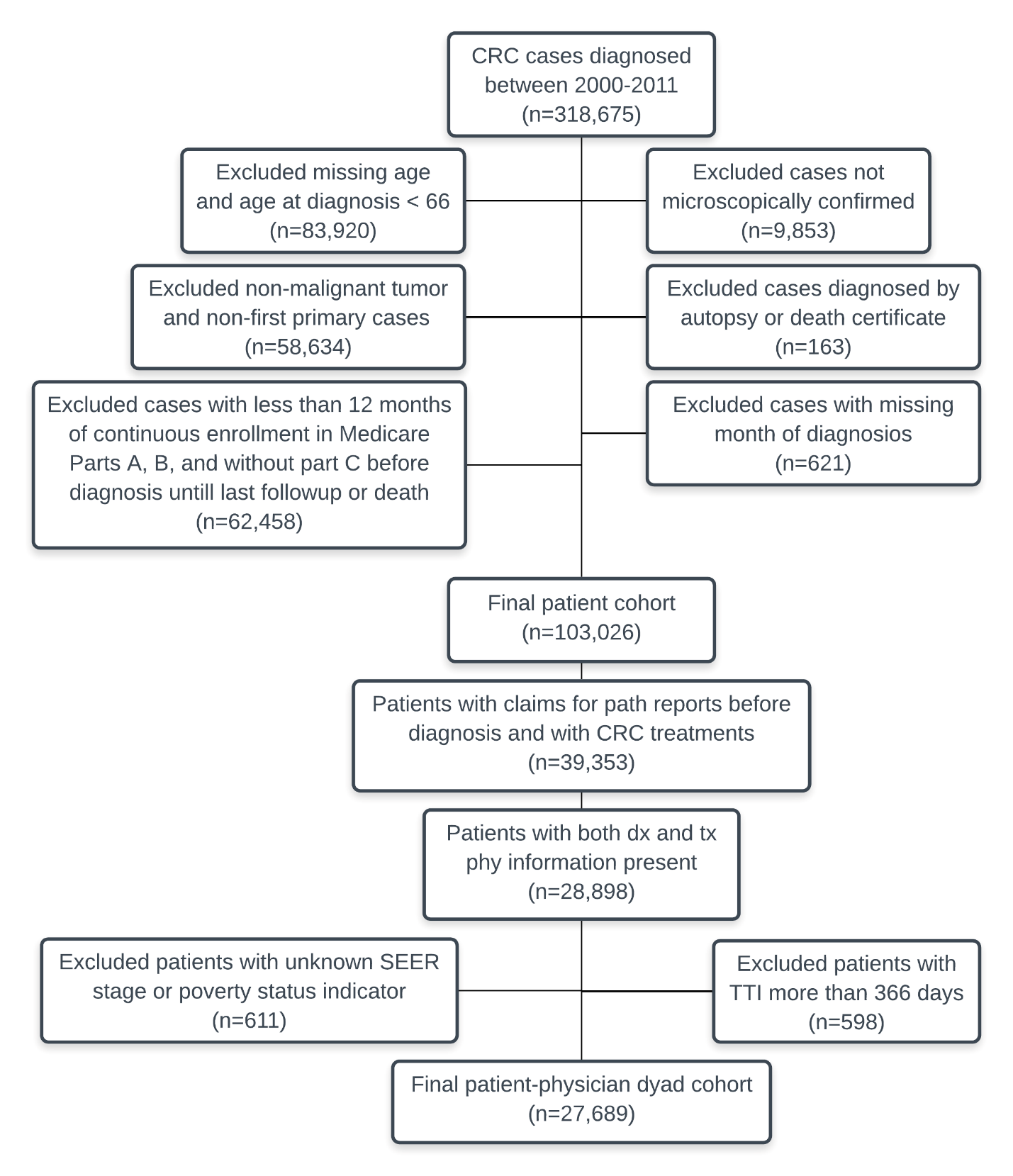
**

**eFigure 2.** Patient-level and Provider-Level Network Case Selection Tree. Flow chart of algorithm development using RECORD (an adapted expansion on STROBE guidelines).

Abbreviations: TTI, time to treatment initiation; CRC, colorectal cancer; DX, diagnosis; TX, treating.

**
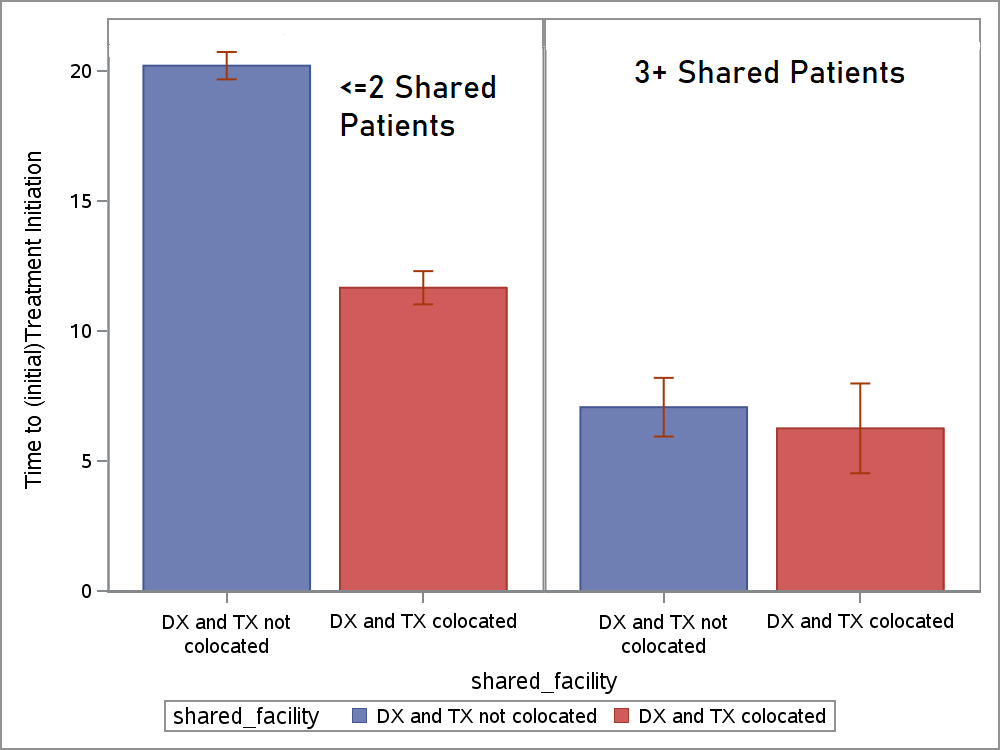
**

**eFigure 3.** Histogram of Diagnosing (DX) Provider-Treating (TX) Provider Dyad by PRF dyad and Time to Treatment (in days). A PRF is associated with shorter time to treatment.

Abbreviations: PR, Patient-Physician Relationship.
